# Removal of cationic and anionic heavy metals from water by 1D and 2D-carbon structures decorated with magnetic nanoparticles

**DOI:** 10.1038/s41598-017-14461-2

**Published:** 2017-10-26

**Authors:** Chella Santhosh, Ravi Nivetha, Pratap Kollu, Varsha Srivastava, Mika Sillanpää, Andrews Nirmala Grace, Amit Bhatnagar

**Affiliations:** 1 Department of Environmental and Biological Sciences, University of Eastern Finland, P.O. Box 1627, FI-70211 Kuopio, Finland; 2 Center for Nanotechnology Research, VIT University, Vellore, 632014 Tamil Nadu India; 3 CASEST, School of Physics, University of Hyderabad, Gachibowli, Hyderabad, 500046 India; 4 Newton Alumnus Researcher- The Royal Society London, Thin Film Magnetism group, Cavendish Laboratory, University of Cambridge, Cambridge, CB3 0HE UK; 5 Laboratory of Green Chemistry, Lappeenranta University of Technology, Sammonkatu 12, Mikkeli, 50130 Finland; 6 Department of Civil and Environmental Engineering, Florida International University, Miami, FL 33174 USA

## Abstract

In this study, cobalt ferrites (C) decorated onto 2D material (porous graphene (PG)) and 1D material (carbon nanofibers (CNF)), denoted as PG-C and CNF-C nanocomposites, respectively, were synthesized using solvothermal process. The prepared nanocomposites were studied as magnetic adsorbents for the removal of lead (cationic) and chromium(VI) (anionic) metal ions. The structural and chemical analysis of synthesized nanocomposites was conducted using different characterization techniques including Brunauer–Emmett–Teller (BET) analysis, field emission-scanning electron microscopy (FE-SEM), Fourier-transform infrared spectroscopy (FTIR), high resolution-transmission electron microscopy (HR-TEM), vibrating sample magnetometer (VSM), X-ray diffraction (XRD), and X-ray photoelectron spectroscopy (XPS). Batch mode adsorption studies were conducted with the prepared nanocomposites to examine their maximum adsorption potential for lead and chromate ions. Performance parameters (time, pH, adsorbent dosage and initial ion concentrations) effecting the adsorption capacity of the nanocomposites were optimized. Different kinetic and isotherm models were examined to elucidate the adsorption process. Synthesized nanocomposites exhibited significant potential for the studied metal ions that can be further examined at pilot scale for the removal of metal ions from contaminated water.

## Introduction

The wastewater, discharged from various industries, contain different kinds of pollutants and these toxic pollutants contaminate the fresh water bodies posing a stern risk to the environment and living organisms^1^. Among different types of aquatic pollutants, heavy metals are the most significant ones, because they are very toxic even at very low concentrations and are persistent in the environment thereby, threatening the environment and biota^2,3^. Electroplating industry, tanneries, electronics manufacturing industry, coal-fired power plants and mining operation are key sources of heavy metal pollution in water. Thus, it is essential to treat the industrial effluents containing metals before their discharge to prevent heavy metal pollution of water^4,5^.

Lead (Pb(II)), a potentially toxic metal ion, is discharged into water sources from various industries including automobile batteries, fuels, printing processes, photographic materials, pigments, ceramic and glass^6,7^. Pb(II) is highly toxic to human beings and biota even at trace concentrations. Exposure to high levels of Pb(II) could damage the central nervous system and brain and lead to death^8^. Maximum contaminant level of Pb(II) in drinking water is set at 15 µg L^−1^ by US Environmental Potential Agency (US EPA)^9^. Chromium, another toxic heavy metal pollutant, is mainly present in the effluents of leather tanning, electroplating, metal finishing, textile industries, and chromate preparations^10^. Chromium is found in two oxidation states, + 3 and + 6. As compared to the + 3 oxidation state, + 6 oxidation state of chromium is considered more toxic^10^. World Health Organization (WHO) guideline for Cr(VI) in drinking water is 50 µg L^−1^ ^11^. Exposure to elevated levels of Cr(VI) might lead to gastrointestinal disorders, liver, kidney and lung cancer, cardiovascular shocks, and other health related problems^12^.

Water and wastewater treatment has been widely investigated with available techniques including precipitation, sedimentation, reverse osmosis, ion-exchange, membrane process, electrochemical and adsorption^13^. Among all the mentioned techniques, the adsorption process has been widely explored because adsorption based systems are simple to design, easy to operate, economical and shows higher efficiency towards the removal of various toxic pollutants including metals^14,15^. Activated carbon, a commercially available adsorbent, has been extensively used for the treatment of wastewater^16,17^, but it’s use is sometimes limited due to its high cost. From the past few years, nanomaterials are being explored in water treatment applications owing to their beneficial properties which include higher surface area, enhanced reactivity and increased surface/volume ratio^18^. The use of magnetic nanocomposites can also be beneficial due to the fact that they exhibit better adsorption potential and easy separation/recovery from the aqueous solution after the process^19^.

On the other hand, carbon-based materials with porous structures also find extensive use in many fields such as adsorption, separations, supercapacitors, drug delivery, lithium ion batteries and sensing due to their distinctive properties^20–25^. One dimensional (1D) structures of carbon-based materials such as carbon nanotubes (CNTs), carbon nanofibers (CNFs) and carbon nanowires have been studied as adsorbents for the removal of aquatic pollutants^26–28^. Compared to 1D materials, that exhibit low adsorption potential, research has been directed to 2D materials, which possess higher surface area and show better adsorption potential than 1D materials. Graphene is a 2D material, which is a derivative of the carbon family and can exhibit high porosity. Graphene-based adsorbents have been widely explored in water treatment applications^29–32^. Porous graphene exhibits higher efficiency than pristine graphene because of the presence of porous structures on the graphene sheets which leads to higher surface area, more adsorption sites thus, making it valuable for many applications. In this regard, a robust method for the synthesis of porous graphene (PG) is highly attractive for such kind of applications.

In the present study, cobalt ferrite (CoFe_2_O_4_) nanoparticles, decorated onto two different carbon materials (viz., 1D (carbon nanofibers (CNF)) and 2D (porous graphene (PG)) were synthesized via solvothermal process. The synthesized materials were analyzed by various characterization techniques to understand their physicochemical properties. The synthesized nanocomposites were investigated for the adsorption of Pb(II) (cationic) and Cr(VI) (anionic) metal ions from water. Optimization of experimental conditions (viz., contact time, solution pH, initial adsorbate concentration, adsorbent dosage and temperature) was performed using batch mode analysis. Different models (kinetic and isotherm) were fitted to identify the underlying adsorption mechanism.

## Results and Discussion

### X-Ray diffraction analysis

**Figure 1:**
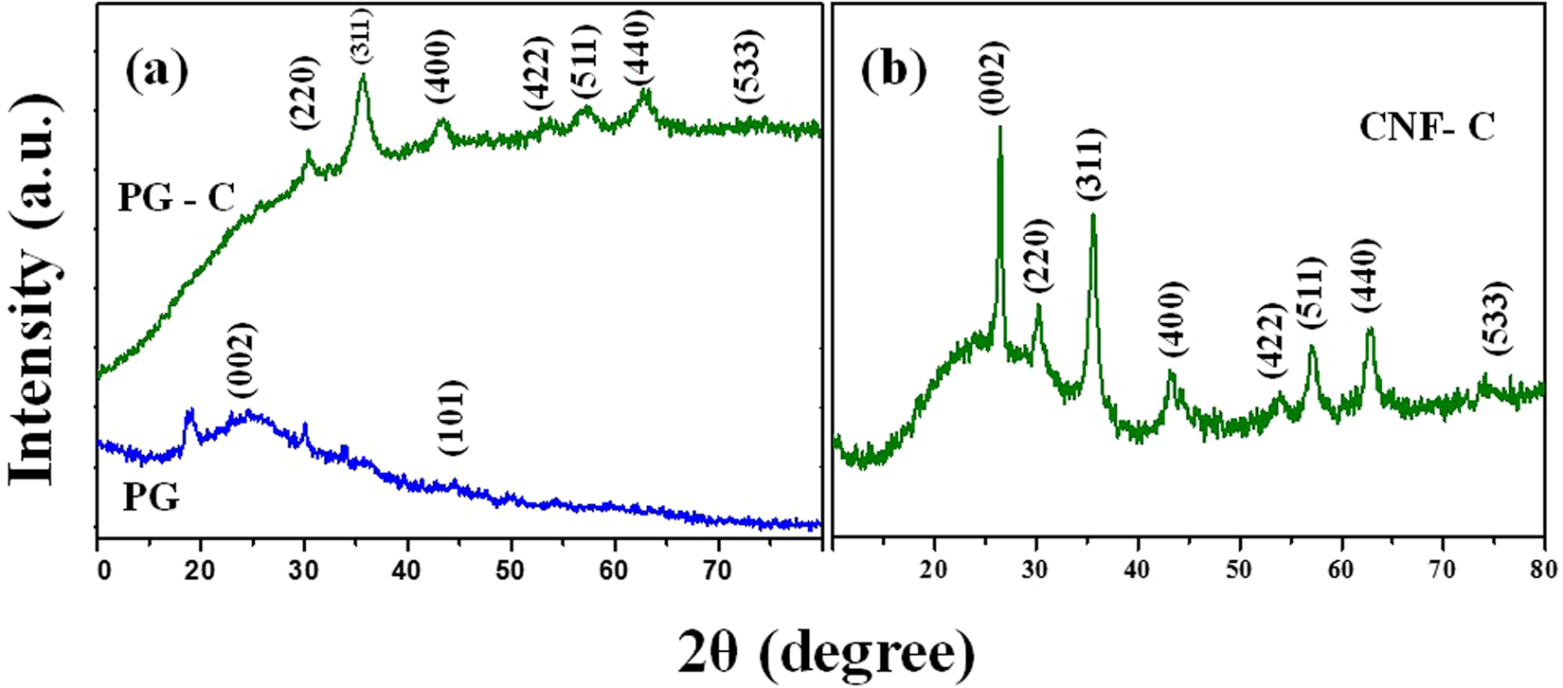
(**a**) XRD analysis of porous graphene (PG) and porous graphene – CoFe_2_O_4_ (PG-C) and (**b**) carbon nanofiber – CoFe_2_O_4_ (CNF-C).

The XRD analyses of PG, PG-CoFe_2_O_4_ (PG-C) and CNF-CoFe_2_O_4_ (CNF-C) nanocomposites are shown in Fig. 1. Fig. 1(a) exhibits the XRD spectra of porous graphene (PG) and PG-C. The intense peaks of (002) and (101) shows the diffraction peaks of porous graphene (PG) and the crystalline planes of (220), (311), (400), (422), (511), (440) and (533) are the diffraction peaks at 2θ values of 30.21°, 35.29°, 43.25°, 53.72°, 57.01°, 62.56° and 74.22°, respectively of PG-C. The obtained peaks of PG-C are ascribed to the spinel-type CoFe_2_O_4,_ which is matching with the standard JCPDS No. 22-1086. Solvothermal technique facilitated the formation of graphene by exfoliation process and further decoration of CoFe_2_O_4_ nanostructures on the layers uniformly, which might be a reason for the disappearance of (002) diffraction peak and similar results have been reported in earlier reports^33^. The XRD spectrum of CNF-CoFe_2_O_4_ composites is shown in Fig. 1(b). The 2θ values of 26.40°, 30.48°, 35.75°, 43.27°, 53.98°, 57.02°, 62.93° and 74.29° are the relevant peaks pertaining to (002), (220), (311), (400), (422), (511), (440) and (533) planes for CNF-C. The peak (002) shows the prominence peak of carbon nanofiber (CNF) present in the nanocomposite, which reveals the presence of spinel structures with Oh7-Fd3m space group.

### Morphological analysis

**Figure 2:**
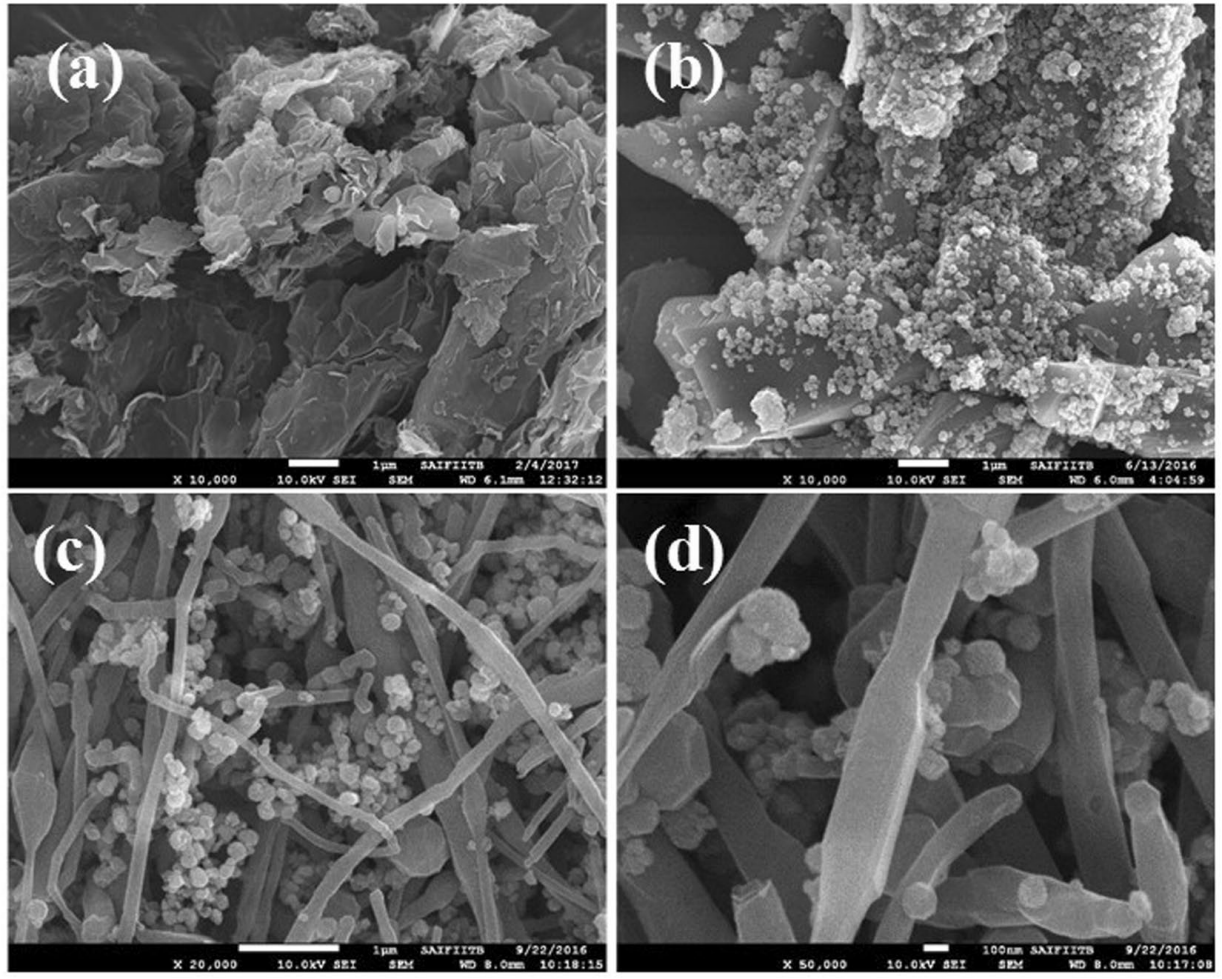
FE-SEM images of (**a**) porous graphene (PG), (**b**) porous graphene - CoFe_2_O_4_ (PG-C), (**c**–**d**) carbon nanofibers - CoFe_2_O_4_ (CNF-C) at different magnifications.

**Figure 3:**
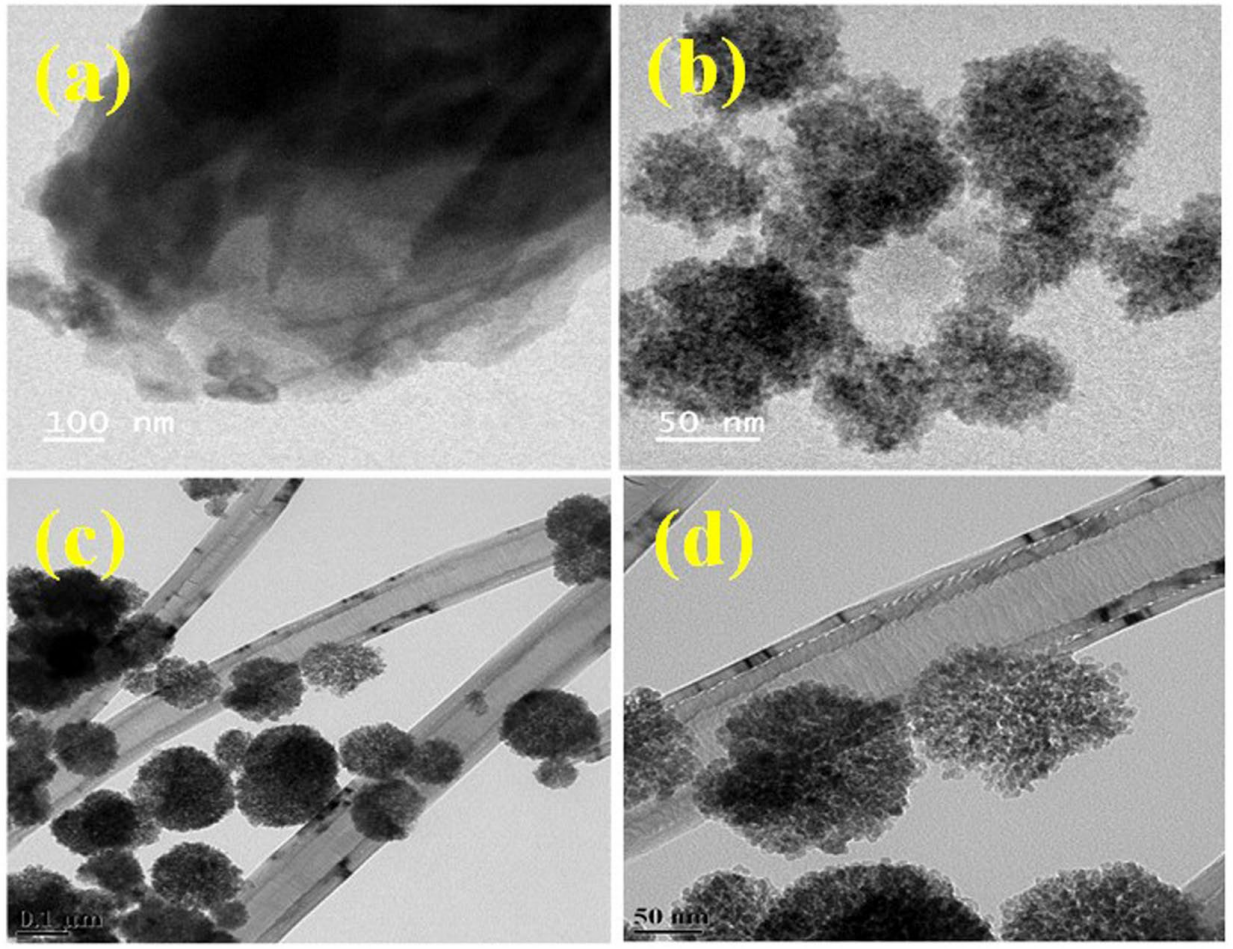
HR-TEM images of (**a**) porous graphene (PG), (**b**) porous graphene - CoFe_2_O_4_ (PG-C), (**c**–**d**) Carbon nanofibers - CoFe_2_O_4_ (CNF-C) at different magnifications.

Figure 2 shows the typical morphology of the synthesized nanocomposites as examined by FE-SEM. As observed from the FE-SEM analysis, CoFe_2_O_4_ particles are homogeneous sphere-shaped particles dispersed on carbon nanofibers and porous graphene sheets. However, the particle clusters were mainly sized between 100–150 nm, but aggregation was also noticed. Structures of the PG-C and CNF-C nanocomposites were also determined by HR-TEM (Fig. 3). Porous structures of nanocomposites were observed in both, PG-C (Fig. 3(b)) and CNF-C (Fig. 3(c) and (d)). The carbon nanofiber diameters varied from 120–150 nm and in the case of CoFe_2_O_4,_ the particles were identified as aggregates of smaller moieties having porous structure of 10–15 nm in size. Flake-like graphene nanosheets and carbon nanofibers were decorated with CoFe_2_O_4_ spheres having an average diameter of 150 nm.

### Magnetic studies

**Figure 4:**
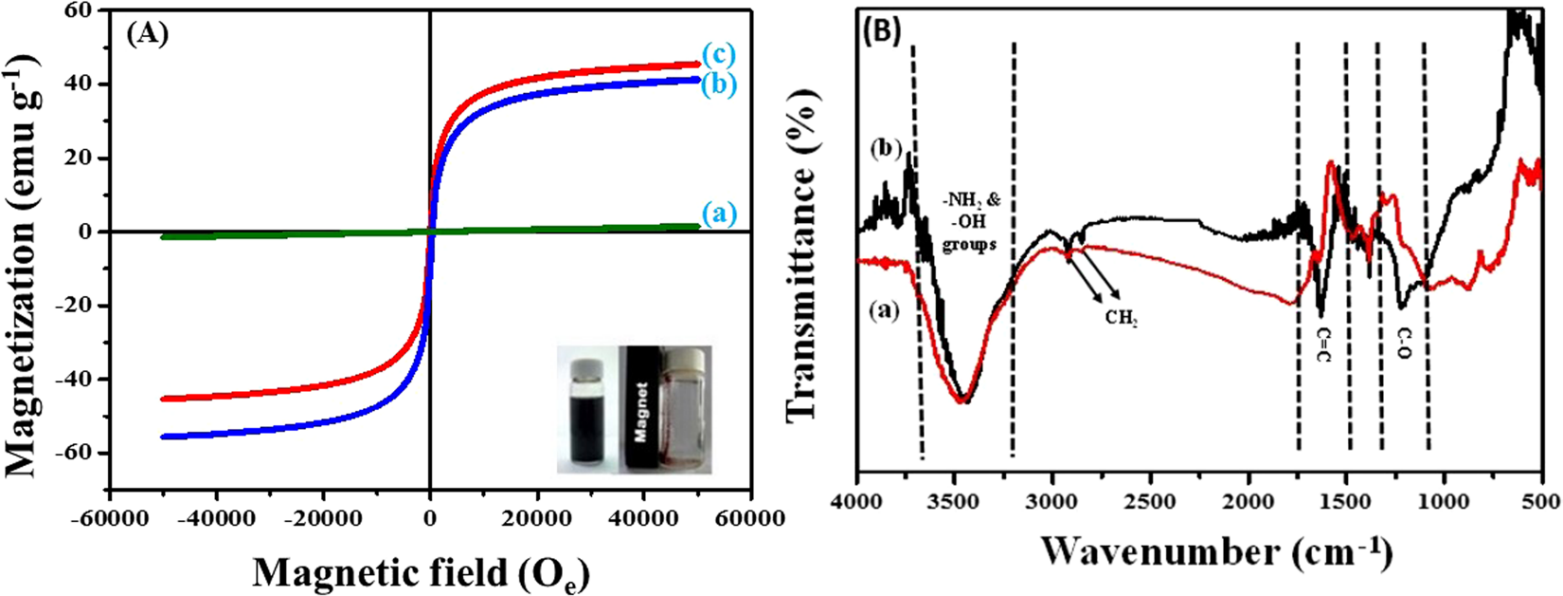
(**A**) Hysteresis (M-H) analysis of (a) porous graphene (PG), (b) porous graphene – CoFe_2_O_4_ (PG-C) and (c) carbon nanofibers – CoFe_2_O_4_ (CNF-C); (**B**) FT-IR analysis of (a) porous graphene – CoFe_2_O_4_ (PG-C) and (b) carbon nanofibers – CoFe_2_O_4_ (CNF-C).

A magnetic sorbent could be used efficiently due to its magnetic properties as it can be recovered back and easily separated from treated water. Magnetic properties of the prepared nanocomposites were measured at magnetic field of −60,000 ≤ H ≤ 60,000 Oe at 27 °C, where H is the magnetic field strength. The corresponding hysteresis loops of PG, PG-C and CNF-C are presented in Fig. 4A, and these loops prove the magnetic properties f the synthesized materials. As compared to PG-C and CNF-C, the saturation magnetization of PG (Fig. 4A) is almost zero, because graphene does not possess any magnetic property, whereas PG-C and CNF-C have the magnetic properties due to the attachment of CoFe_2_O_4_ nanoparticles onto the PG and CNF as can be observed in Fig. 4(A). The CoFe_2_O_4_ nanoparticles which are decorated onto graphene sheets behave as magnetically active layers, that in turn affects the magnetic properties of synthesized nanocomposites, which was also confirmed by FE-SEM and HR-TEM images (Figs 2 and 3). A high value of saturation magnetization of the prepared materials, PG-C and CNF-C, could further facilitate the re-usage of the adsorbent.

### FT-IR analysis

FTIR spectra of PG-C and CNF-C recorded within the range of 400–4000 cm^−1^ is presented in Fig. 4(B). The broad peak near 3466 cm^−1^ confirms the existence of –NH_2_ and –OH groups, which are present on the surface of CoFe_2_O_4_ nanoparticles as well as absorbed water molecules. The characteristic peaks at 2926 and 2836 cm^−1^ could be attributed to the asymmetric and symmetric CH_2_ stretching, respectively. The sharp peaks at 1648 and 1255 cm^−1^ are associated with C = C, -C–O, respectively^34–36^. The peak at 810 cm^−1^ corresponds to the O-H vibration. The band at 580 cm^−1^ might be attributed to the occurrence of ferrites in the synthesized magnetic nanocomposites.

### XPS and BET analysis

**Figure 5:**
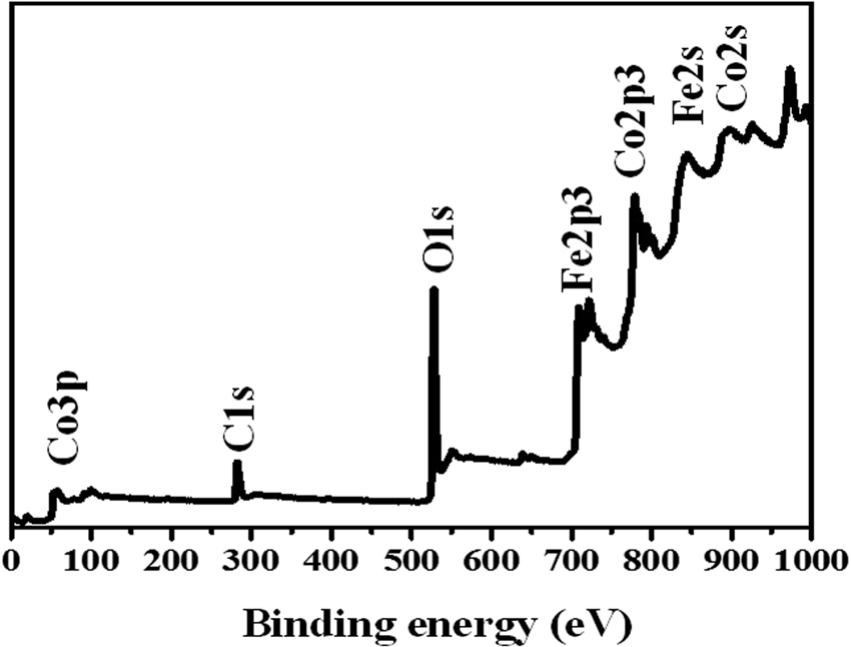
Wide scan XPS spectra of porous graphene – CoFe_2_O_4_ (PG-C) nanocomposite.

The chemical composition of PG-C and CNF-C was further explored using X-ray photoelectron (XPS) spectroscopy. The binding energy at 284.6 eV pertaining to C1s was used as reference. As can be observed in Fig. 5, all the relevant peaks of C1s, Co 2p, O1s and Fe 2p were distinct. To determine the porous nature of PG-C and CNF-C, N_2_ adsorption–desorption isotherm was examined (Supplementary Fig. S1). In accordance with IUPAC classification, the N_2_ gas adsorption–desorption isotherm exhibits type IV curve and H3 hysteresis loop. The obtained isotherm (Fig. S1) is a clear evidence of presence of mesopores in PG-C and CNF-C^37,38^. Type H3 hysteresis reveals the arbitrary dispersal of pores and interconnection. Such pore characteristics profoundly dominate the desorption isotherm rather than adsorption isotherm owing to diverse behavior of adsorption and desorption isotherm with respect to pore network at a relative pressure of 0.45 (for N_2_ at 77 K). Both PG-C and CNF-C exhibit obvious hysteresis loops when P/P_0_ range from 0.4 to 1.0 nm. The specific surface area of PG-C and CNF-C was determined as 154.54 and 45.74 m^2^ g^−1^, respectively, using the Brunauer-Emmett-Teller (BET) analysis. The Barrett-Joyner-Halenda (BJH) desorption average pore diameter was calculated as 8.8 and 16 nm for PG-C and CNF-C, respectively, with a broad pore size distribution as presented in Supplementary Fig. S1.

### Effect of contact time

The kinetics of Pb(II) adsorption onto PG-C and CNF-C was explored and the results are provided in Supplementary Fig. S2(a). Equilibrium of Pb(II) was achieved in 120 min in case of PG-C and CNF-C. The adsorption potential of PG-C for Pb(II) ions was found to be higher than CNF-C. Based on the obtained results, 140 min was fixed as the equilibrium time for further investigation of Pb(II) adsorption by synthesized nanocomposites. As observed by BET analysis, PG-C possesses high surface area (154.54 m^2^ g^−1^) as compared to CNF-C (45.74 m^2^ g^−1^), which might be one of the reasons for the higher adsorption capacity of PG-C for Pb(II) ions. Kinetics studies were also conducted for Cr(VI) ions adsorption by PG-C and CNF-C (Supplementary Fig. S2(c)). The adsorption was found to increase up till 160 min. Beyond 160 min, no further increase in the adsorption was observed, and hence 160 min was selected as optimum contact time for Cr(VI) adsorption by the two nanocomposites.

**Figure 6:**
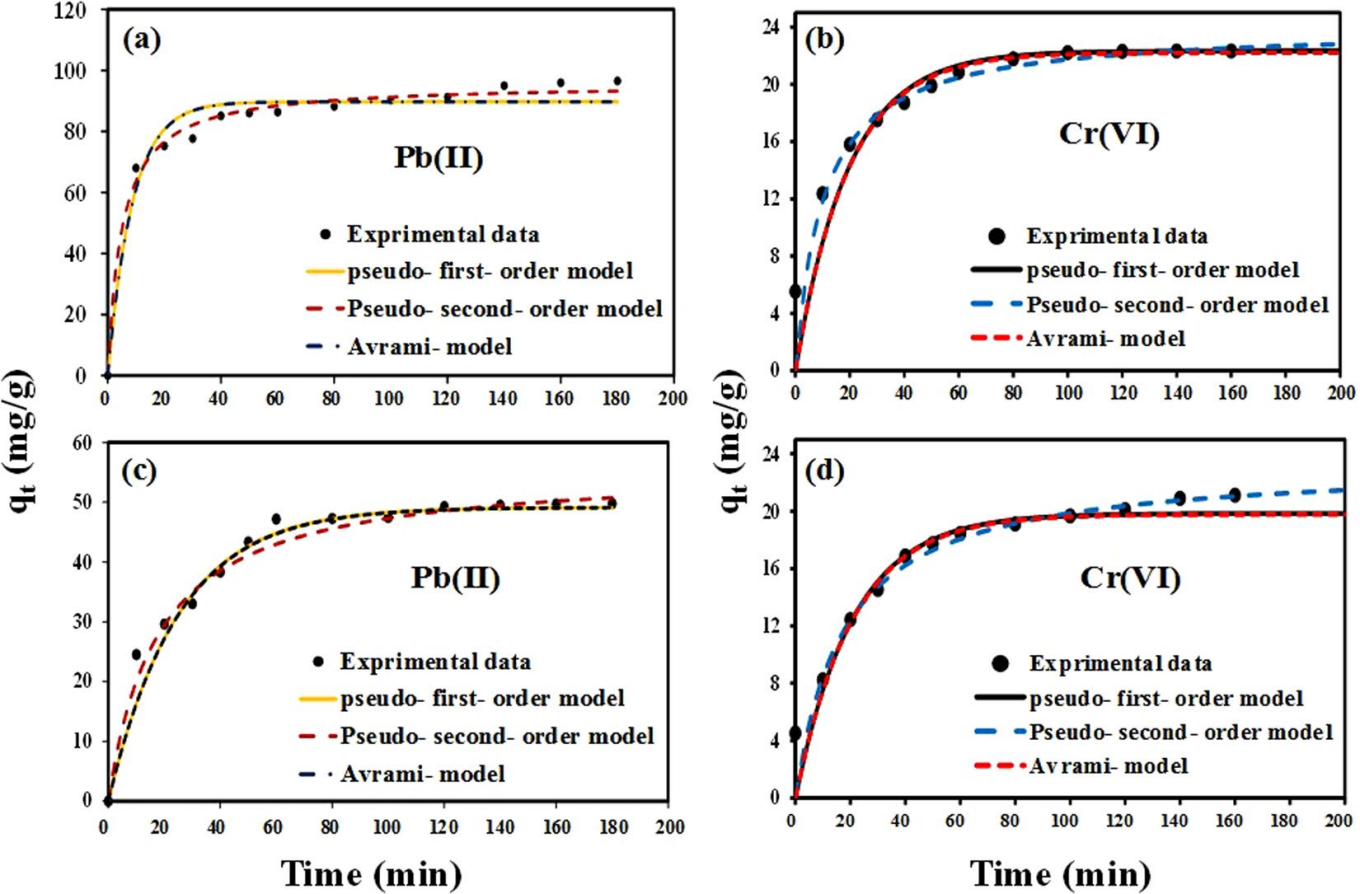
Adsorption kinetics of Pb(II) and Cr(VI) ions adsorption by (**a**,**b**) PG-C and (**c**,**d**) CNF-C nanocomposites and fitting of different kinetic models to the experimental data.

Various kinetic models viz., pseudo-first-order, pseudo-second-order, Avrami and intra-particle diffusion models (Eqs 1–4)^39–42^ were tested to define the adsorption kinetics of Pb(II) and Cr(VI) ions adsorption onto the nanocomposites.

1 $${q}_{t}={q}_{e}(1-{e}^{-{k}_{1}t})$$

2 $${q}_{t}=\frac{{k}_{2}{q}_{e}^{2}t}{1+{k}_{2}{q}_{e}t}$$

3 $${q}_{t}={q}_{e}(1-{e}^{(-{({K}_{AV}t)}^{n}AV)})$$

4 $${q}_{t}={K}_{p}{t}^{\frac{1}{2}}+I$$

where, *q*_*e*_ and *q*_*t*_ (mg g^−1^) denotes the adsorption capacities at equilibrium and at time *t* (min), *k*_1_ (min^−1^) and *k*_2_ (g mg^−1^min^−1^) are the pseudo-first and pseudo-second-order rate constants. K_AV_ (min^−1^) is the Avrami constant; K_p_ and I are the intra-particle diffusion constant and intercept, respectively. The kinetic models were fitted to the experimental data and the modeling results are shown in Fig. 6 and Supplementary Table S1.

The experimental values were found to be closer to the theoretical q_e_ values for pseudo-second-order model giving high correlation coefficient (R^2^) and lower RMSE (root mean square error) values (Fig. 6(a–d)). It was noticed that the experimental values obtained for the adsorption of Pb(II) and Cr(VI) ions onto PG-C and CNF-C did not fit well to the Avrami model. Three distinctive phases were observed when the kinetics was modelled using intra-particle diffusion model (Supplementary Fig. S3) which indicates that apart from the pore diffusion mechanism, there could be other mechanisms involved governing the rate controlling step in the adsorption phenomenon^43^. The estimated values, attained from the applied kinetic models, are presented in supplementary material (Table S1).

### Effect of pH and adsorbent dosage

The adsorption potential of the synthesized nanocomposites was also examined as a function of pH in view of its influence on the adsorption process and also on the speciation of metals in the solution. The pH variation was studied from pH 2.0 to 8.0 for Pb(II) ions adsorption onto both the synthesized nanocomposites (PG-C and CNF-C). At acidic pH (2.0–3.0), the adsorbents exhibited low adsorption capacity for Pb(II) ions which could be attributed to the excess of hydrogen ions in solution that can compete with Pb(II) ions for active sites. At lower pH, adsorbent surface could be saturated with hydronium ions and hence, populated with protonated sites, rendering the surface charge net positive, which will replace the positively charged Pb(II) ions. Hence, the adsorption capacity was found to decrease at lower pH. Whereas at higher pH (4.0–8.0), the adsorbent surface would become negatively charge, and as a result, the positively charged Pb(II) ions tend to get attracted towards the negative charged functional groups present on the synthesized nanocomposites via electrostatic attraction. It can be seen in Fig. S2(b) (Supplementary material) that the adsorption increased around pH 4.0, and was found to be maximum at pH 7.0. Beyond pH 7.0, no significant increase in the Pb(II) adsorption was observed, which can be due to the precipitation of Pb(II) as Pb(OH)_2_. Thus, the maximum adsorption efficiency of synthesized nanocomposites for Pb(II) ions was achieved in the pH range between 4.0–7.0.

Chromium(VI) occurs in different ionic forms (HCrO_4_^−^, CrO_4_^2−^ and Cr_2_O_7_^2−^) at different pH ranges^44^. HCrO_4_^−^ is the main form of Cr(VI) at pH < 6.0, and it converts into CrO_4_^2−^ with the increase in pH ( > 6.8)^44^. Cr(VI) adsorption by PG-C and CNF-C nanocomposites was also investigated to determine the change in adsorption efficiency as a function of pH (2.0–10.0) (Supplementary Fig. S2(d)). The adsorption capacity of Cr(VI) was highly influenced by the change in solution pH. Maximum Cr(VI) adsorption occurred in the acidic pH range and decreased at neutral and basic solution pH. Abundance of hydroxyl groups at higher pH hindered the adsorption of chromate ions by PG-C and CNF-C nanocomposites^45^.

Adsorbent dose is also critical factor that needs to be studied to optimize the adsorption process. The influence of adsorbent dosage on Pb(II) adsorption was conducted by fluctuating the dosage of PG-C and CNF-C from 0.5 to 3.0 g L^−1^ (Supplementary Fig. S4(a)). As the adsorbent dosage was increased from 0.5–3.0 g L^−1^, adsorption of Pb(II) ions increased which might be attributed to the available of extra active sites on the surface of the nanocomposites. On further increasing the adsorbent dose (from 1.5 to 3.0 g L^−1^), the previously available active sites became saturated resulting in the lower Pb(II) ions adsorption by nanocomposites. The optimum adsorbent dose for PG-C and CNF-C for Pb(II) was determined to be 1.0 and 1.5 g L^−1^ with 99.8% and 48% of adsorption, respectively. Cr(VI) adsorption by PG-C and CNF-C was investigated at dosages ranging from 0.25 to 2.0 g L^−1^ (Supplementary Fig. S4(b)). An increase in the removal efficiency of Cr(VI) ions was observed (from 60 to 100%) as the dosage was raised from 0.25 to 0.5 g L^−1^ for PG-C. Whereas in case of CNF-C, the removal efficiency increased from 50 to 100% with an increase in dosage from 0.25 to 1.0 g L^−1^. Hence, the optimum adsorbent dosage for PG-C and CNF-C for Cr(VI) was set as 0.5 and 1 g L^−1^ for further investigation.

### Adsorption isotherms

**Figure 7:**
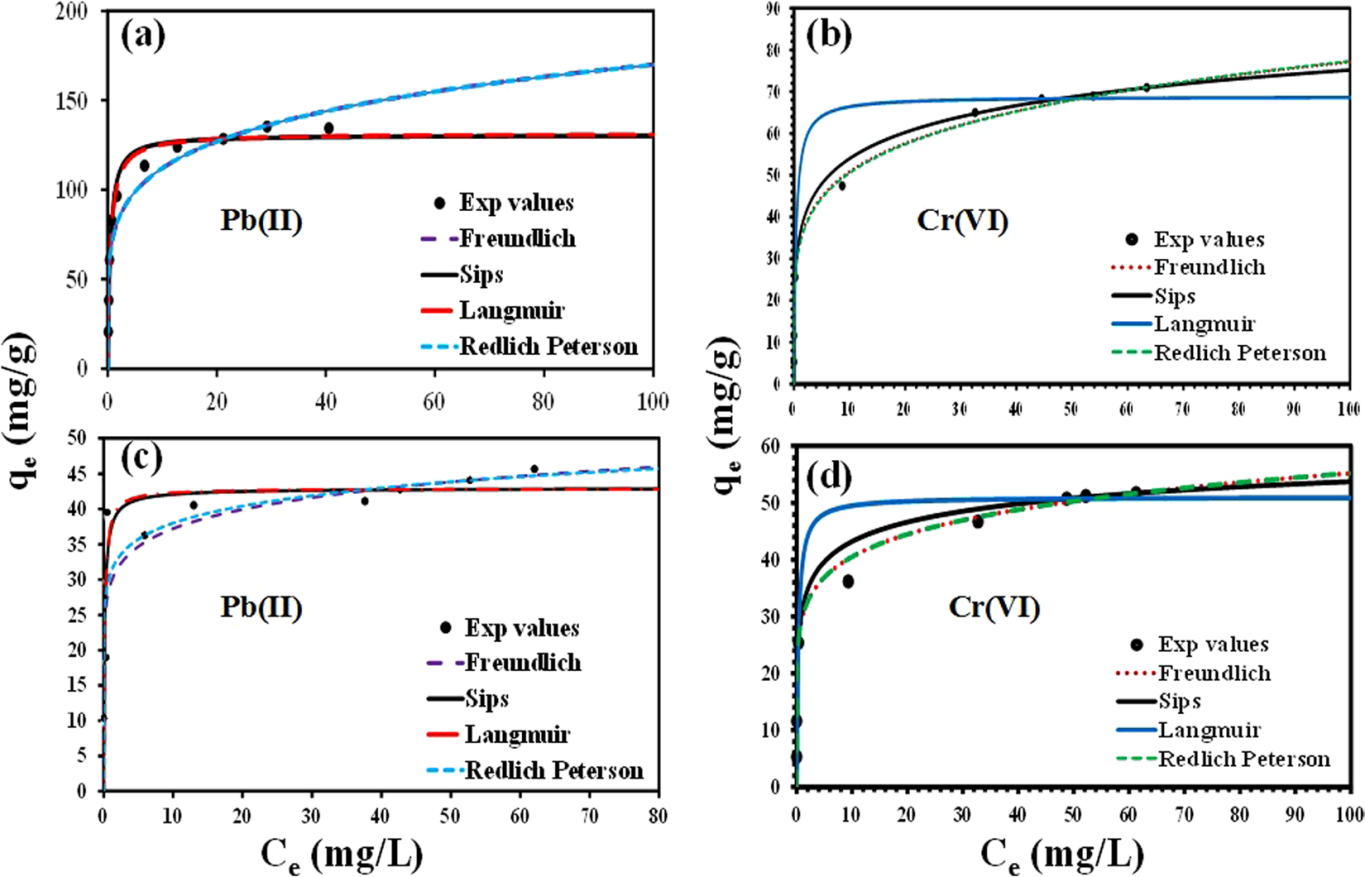
Adsorption isotherms of Pb(II) and Cr(VI) ions adsorption by (**a**,**b**) PG-C and (**c**,**d**) CNF-C nanocomposites and fitting of different isotherm models to the experimental data.

The adsorption was also examined as a function of adsorbate concentration [Pb(II) (C_i_ = 10–100 mg L^−1^) and Cr(VI) (C_i_ = 2.5–100 mg L^−1^)], and the results are presented in Fig. 7. A maximum uptake capacity of 131.40 and 42.90 mg g^−1^ was achieved for Pb(II) ions onto PG-C and CNF-C nanocomposites, respectively, at room temperature (25 °C). Whereas for the Cr(VI) ions, the maximum uptake capacity was recorded as 68.85 and 51.07 mg g^−1^ onto PG-C and CNF-C, respectively, at 25 °C. A difference in the adsorption capacity between two nanocomposites is observed because the former nanocomposite has vastly porous structure and higher surface area, which leads to the higher adsorption of the target pollutants. It is evident from Fig. 7 that there is an initial increase in the adsorption capacity of nanocomposites for Pb(II) and Cr(VI), which might be attributed to the availability of plenty of vacant sites in the beginning of adsorption. The equilibrium was attained after 120 min, indicating the saturation of available sites and unavailability of further vacant sites for adsorption onto PG-C and CNF-C nanocomposites. The decrease of available adsorption sites on the surface of PG-C and CNF-C results in the decreased driving force and prolongation of the equilibration level and decreased rate of adsorption.

To validate the adsorption equilibrium results, Langmuir, Freundlich, Redlich–Peterson, and Sips models were fitted to the experimental results. The Langmuir model holds true for monolayered adsorption onto homogenous adsorbent’s surface^46^ whereas Freundlich is valid if the adsorption is multilayer on heterogeneous surface of the adsorbent^47^. A combination of Langmuir and Freundlich is Sips model and the Redlich-Peterson model includes three parameters applied for both homogeneous or heterogeneous systems^48,49^. The non-linear forms of Langmuir, Freundlich, Sips and Redlich-Peterson models are given below (Eqs 5–8).

5 $${q}_{e}=\frac{{q}_{m{K}_{L}{C}_{e}}}{1+{K}_{L}{C}_{e}}$$

6 $${q}_{e}={K}_{F}{C}_{e}^{\frac{1}{n}}$$

7 $${q}_{e}=\frac{{q}_{m{({K}_{S}{C}_{e})}^{m}}}{1+{({K}_{S}{C}_{e})}^{m}}$$

8 $${q}_{e}=\frac{{K}_{RP}{C}_{e}}{1+{a}_{RP}{C}_{e}^{\beta }}$$

where, amount of target heavy metal ions adsorbed by PG-C and CNF-C at equilibrium and maximum adsorption capacity are denoted by *q*_*e*_ (mg g^−1^) and *q*_*m*_ (mg g^−1^), respectively; and equilibrium concentration in solution is *C*_*e*_ (mg L^−1^). The Langmuir constant and Freundlich equilibrium constant are denoted by *K*_*L*_ (L mg^−1^) and *K*_*F*_ (mg g^−1^) (L mg^−1^)^1/n^. The symbol ‘*n*’ is the Freundlich equilibrium exponent. The affinity constant in Sips model is denoted by K_S_ (L mg^−1^); and Redlich-Peterson constants are denoted by *K*_*RP*_ (L g^−1^) and *a*_*RP*_ (L mg^−1^). The results of isotherm modeling of Pb(II) ions adsorption onto PG-C and CNF-C nanocomposites are given in Supplementary Table S2 and Fig. 7(a,c). Isotherm modeling results of Cr(VI) ions adsorption onto PG-C and CNF-C nanocomposites are presented in Supplementary Table S2 and Fig. 7(b and d).

Langmuir model provided the best fit with R^2^ (regression coefficient) values of 0.987 and 0.985 and lower RMSE values (14.75 and 5.19) for Pb(II) ions adsorption onto PG-C and CNF-C nanocomposites. The good fitting indicated a homogenous adsorption onto the surface of synthesized nanocomposites. For Cr(VI) ions, higher values of R^2^ (0.990 and 0.989) and lower RMSE values (7.84 and 5.87) were obtained with Langmuir model, which is better fit as compared with the other isotherm models. To analyze the favorability of adsorption, *R*_*L*_ (dimensionless constant, Eq.(9)) was calculated which would indicate the feasibility of metal ions adsorption onto synthesized nanocomposites^50^:

9 $${R}_{L}=\frac{1}{1+{K}_{L}{C}_{i}}$$

where *K*_*L*_ is the Langmuir constant and initial concentration of metal ions is denoted by *C*_*i*_. Adsorption is regarded as favorable if *R*_L_ < 1, and adsorption is considered as unfavorable if *R*_*L*_ > 1. The *R*_*L*_ values indicates the favorability of adsorption of investigated heavy metal ions viz., (Pb(II) and Cr(VI)) onto synthesized PG-C and CNF-C nanocomposites (Supplementary Table S2).

In general, the stacking process of CoFe_2_O_4_ within the graphene sheets reduces van der Waals forces, thereby preventing the aggregation factor. In addition to this, PG-C did not exhibit pore blockage phenomenon during loading and uniform anchoring of CoFe_2_O_4_ particles on the surface of porous graphene sheets. Therefore, the synthesized nanocomposites exhibit considerable adsorption affinity for studied heavy metal ions. Additionally, the synthesized CoFe_2_O_4_ nanoparticles are also porous in nature. Hence the composites of graphene and CoFe_2_O_4_ enhanced the overall surface area of the synthesized material in turn providing enormous active sites for adsorption, whereas carbon nanofibers nanocomposite (CNF-C) possess less surface area and thus, exhibited lower adsorption capacity for target pollutants.

### Thermodynamic parameters

The thermodynamic feasibility of the adsorption process was investigated to elucidate the nature of adsorption process. The following equations were used for the thermodynamic calculations^51^.

10 $${\rm{\Delta }}{G}^{^\circ }=-RT\,\mathrm{ln}\,{k}_{0}$$

11 $${\rm{\Delta }}{G}^{^\circ }\,={\rm{\Delta }}H^\circ -T{\rm{\Delta }}S^\circ $$

where, gas constant (8.314 J mol^−1^ K^−1^) is denoted by *R*, and T is the temperature in Kelvin. Thermodynamic equilibrium constant related to Langmuir constant (*K*_*L*_) is denoted by *k*_0_,_,_ *ΔG*° is standard Gibb’s free energy (kJ mol^−1^), *ΔH*° and *ΔS*° represent the standard enthalpy (kJ mol^−1^) and standard entropy (J mol^−1^ K^−1^), respectively.

The change in free energy (*ΔG*°) was calculated and given in Supplementary Table S3. In case of both the nanocomposites, the values of *ΔG*° were negative, which suggests that the reaction is spontaneous. The same observation is reported by other researchers^52^. A positive value of *ΔH*° confirms endothermic nature and a positive value of *ΔS*° indicates the affinity of the adsorbents towards the Pb(II) and Cr(VI) ions^53^.

### Desorption and regeneration studies

**Figure 8:**
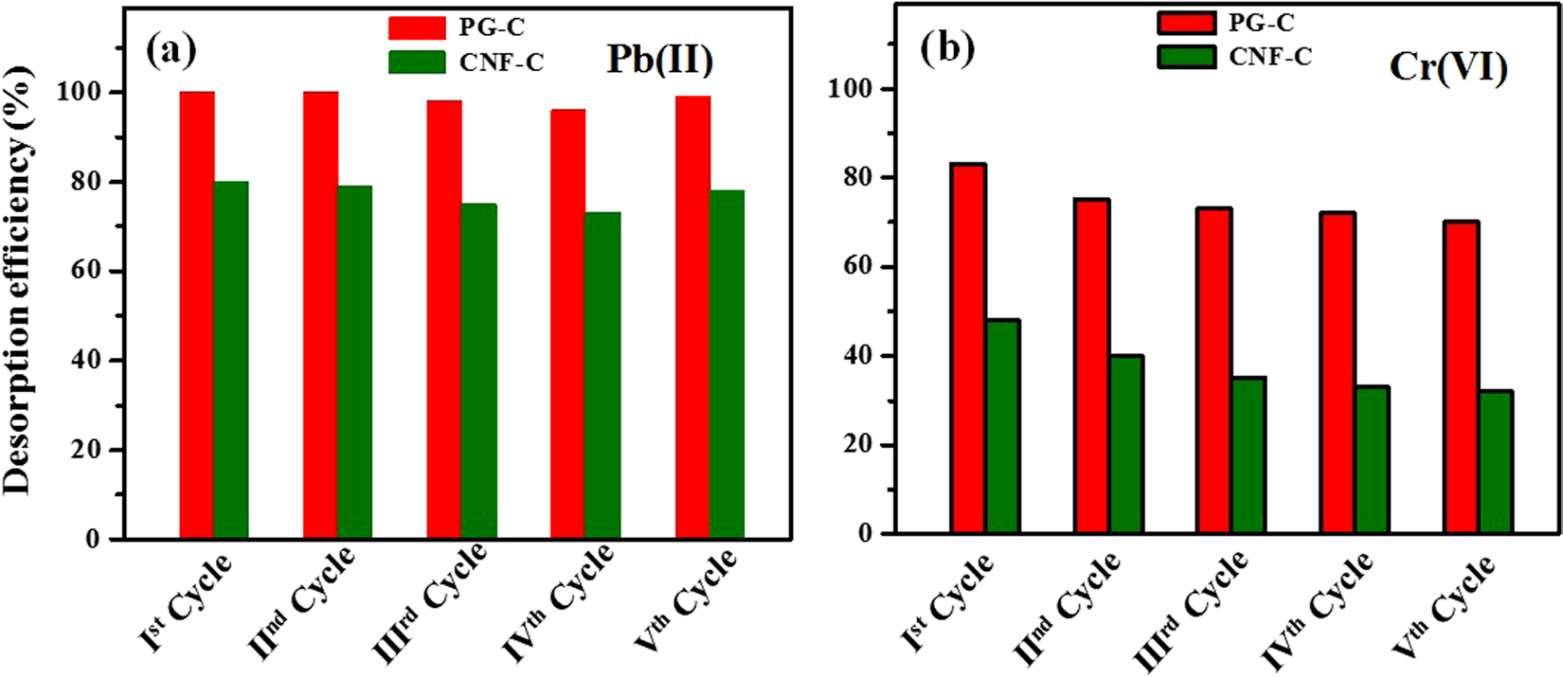
Desorption studies of synthesized nanocomposites for (**a**) Pb(II) and (**b**) Cr(VI) ions.

Regeneration studies are important to be considered in adsorption to make the process economically feasible. Most of the cationic pollutants tend to desorb with the acidic eluents such as HCl, HNO_3_ and H_2_SO_4_. In the case of anionic pollutants, alkaline eluents, such as NaOH, have been generally used as desorbing agents. Metal-loaded PG-C and CNF-C were agitated with 10 mL of HCl (0.1 M) for Pb(II) and NaOH (0.1 M) for Cr(VI) respectively, at 80 rpm for 3 h at 25 °C. The nanocomposites (CNF-C and PG-C) were separated by applying magnetic field and the supernatant solution was filtered with 0.42 µm membrane filters. The filtrate was investigated for residual metal ion concentration and the metal-desorbed PG-C and CNF-C were used for another adsorption cycle. The adsorption-desorption cycle was carried out upto five cycles to access the regeneration efficiency of the nanocomposites. PG-C and CNF-C nanocomposites exhibited desorbing capacity of 95% to 75% for five cycles in case of Pb(II) (Fig. 8a). For Cr(VI), the desorption capacity was observed as 75–80% for PG-C and 35–50% for CNF-C (Fig. 8b). The regeneration studies revealed that the synthesized adsorbents had the potential to be reused for multiple cycles.

## Conclusions

CNF-C (1D) and PG-C (2D) (novel ferrite-based graphene nanocomposites) were synthesized successfully via solvothermal route and further investigated for the adsorptive removal of lead and chromate ions from water. Kinetic and isotherm studies revealed that pseudo-second-order and Langmuir models, respectively explained the sorption mechanisms for both the studied pollutants on CNF-C and PG-C. The adsorption capacity of PG-C and CNF-C for Pb(II) ions was found to be 131.40 and 42.90 mg g^−1^, respectively. In case of Cr(VI) ions, PG-C and CNF-C exhibited adsorption capacity of 68.85 and 51.07 mg g^−1^, respectively. Thermodynamic analysis suggested that the reaction was feasible, spontaneous and endothermic in nature. Lead adsorption was found to be low at acidic pH, and reached a maximum at pH 4.0–7.0. Cr(VI) ions adsorption was higher at acidic pH, and decreased with the increase in pH. Desorption experiments revealed the stable reusable capacity of the adsorbents upto five cycles.

## Experimental Details

### Synthesis of porous graphene (PG)

The synthesis of graphene oxide (GO) was done using natural graphite as a precursor material via modified Hummers method. The procedure, by which synthesis of GO was conducted, is reported and discussed in our previous publication^33^. Porous graphene was prepared via thermal and acid treatment^54^. In brief, KMnO_4_ and GO (ratio 1:2) were added to Milli-Q water, followed by ultra-sonication of the solution for 2 h. Next, the resultant mixture was kept for heating in microwave oven at 700 W for 5 min. After heating, Milli-Q water and hydrazine hydrate (ratio 1:5) were transferred to a round bottom flask and refluxed at 100 °C for 24 h. Then the mixture was washed with 1:1 (v/v) oxalic acid and hydrochloric acid, and next with ethanol and water several times. Finally, the obtained material was kept for drying in a vacuum oven. Henceforth, the synthesized product is denoted as porous graphene (PG).

### Synthesis of PG and CNF based CoFe_2_O_4_ nanocomposites

**Figure 9:**
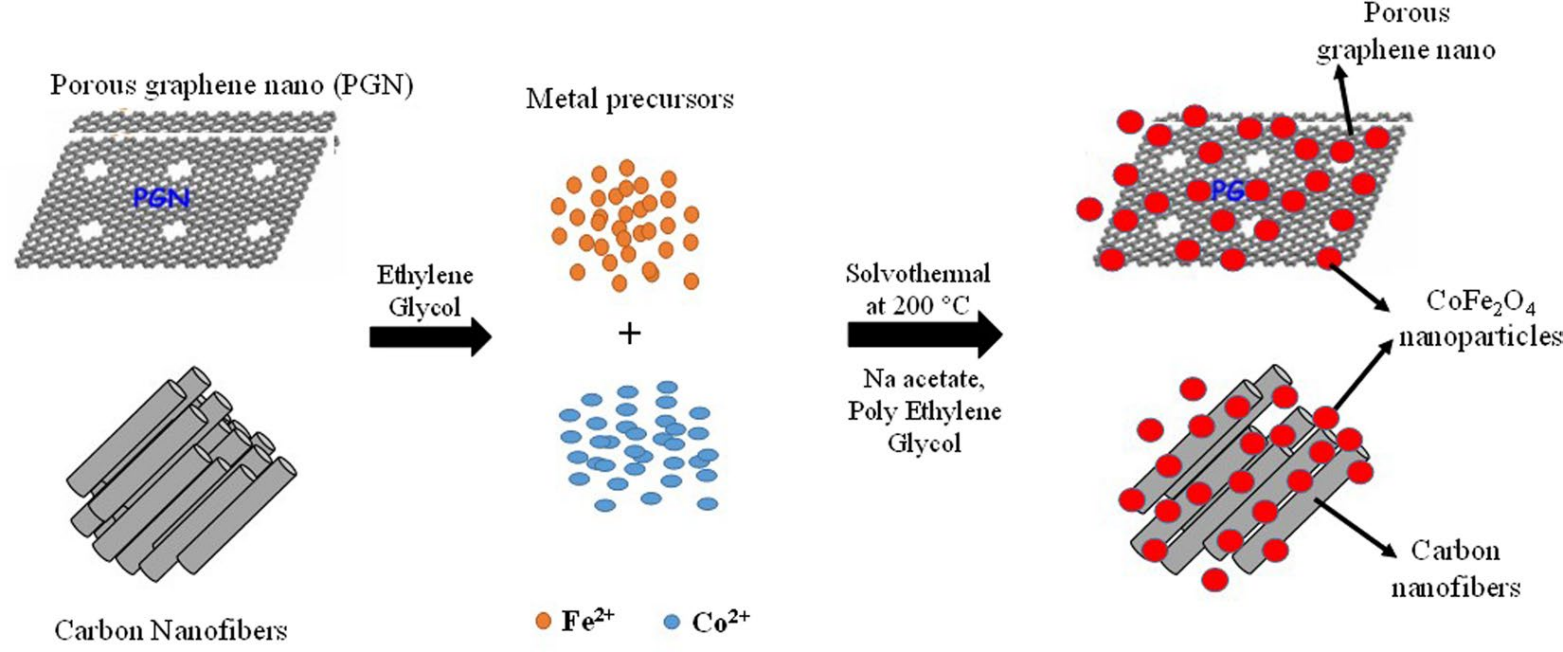
Schematic representation of synthesis process for PG-C and CNF-C nanocomposites.

Cobalt ferrites with 2D (PG) and 1D (CNF) nanocomposites were synthesized via solvothermal process. Briefly, 300 mg of PG and CNF (purchased commercially from Sigma Aldrich (Product number: 719803)) were added to 150 mL of ethylene glycol, containing ferric chloride and cobalt chloride, followed by ultrasonication for 2 h. Further, polyethylene glycol (3 g) and sodium acetate (10 g) were added to the mixture and stirred for 30 min. Then the mixture was autoclaved at 200 °C for 10 h. Finally, the mixture was rinsed with ethanol and DI water and kept for drying in vacuum oven at 45 °C overnight. The pictorial representation of synthesis process of nanocomposites is shown in Fig. 9.

### Measurements and characterization

The crystalline nature of the synthesized nanocomposites was confirmed using an X-ray Diffraction (XRD) (Cu Kα = 1.5406 Å) (Rigaku Miniflex) over 2θ range from 10–80 °C. The morphology of the composites was obtained with FE-SEM, (JSM-7600 F). High-resolution Transmission Electron Microscope (HR-TEM) (FEI-Technai G2, F30 operated at 300 kV) was used to determine the shape and size of the nanocomposites. Physical adsorption of nitrogen (ASAP 2020 Micrometrics instrument) was used to determine the porosity of the samples. Multipoint BET method was used to measure the specific surface area of the synthesized nanocomposites. Pore size distribution and total pore volume were determined by the Brunauer Joyner–Hallenda (BJH) method. Vibrating Sample Magnetometry (VSM) technique was used to measure the magnetic properties of the synthesized nanocomposites using Lake Shore VSM system (Model 7410-S). FT-IR analysis was performed with FT-IR imaging microscope (Bruker Hyperion 3000 microscope, Germany). The Thermo Scientific Multilab 2000 spectrometer was used to conduct X-ray Photo Spectroscopy (XPS) analysis. Elemental analysis was performed with the CHNOS Vario EL cube analysis.

### Batch adsorption studies

Analytical grade salts of Pb(NO_3_)_2_ and K_2_Cr_2_O_7_ were used to prepare stock solutions of Pb(II) and Cr(VI). Adsorption parameters were investigated in batch mode. The experimental conditions including contact time (0–180 min), initial metal ion concentration (2.5–100 mg L^−1^), pH (2.0–8.0) and temperature (25–45 °C) were optimized to achieve maximum adsorption capacities for the investigated adsorbates. The pH of the suspension was adjusted using 0.1 M HCl or NaOH solutions. After shaking for a predetermined time, the adsorbent-adsorbate solutions were centrifuged followed by filtration using 0.42 µm cellulose nitrate membrane filters. The concentration of Pb(II) ions in the solution was quantified using inductively coupled plasma emission spectrometry (ICP-OES: iCAP – 6300, Thermo Electron Corporation). The concentration of Cr(VI) was analyzed using UV-spectrophotometric method with an absorbance wavelength of 540 nm^55^.

The percentage adsorption and equilibrium capacity of Pb(II) and Cr(VI) were calculated using Eqs (12 and 13):

12 $$Adsorption\,( \% )=\frac{{C}_{i}-{C}_{e}}{{C}_{i}}\times 100$$

13 $${q}_{e}=\frac{({C}_{i}-{C}_{e})}{m}V$$

where, *C*_*i*_ and *C*_*e*_ denotes the initial and final (equilibrium) metal ions concentration (mg L^−1^); *q*_*e*_ is the equilibrium adsorption capacity in (mg g^−1^); *V* is the volume of solution (L) and *m* is the weight of adsorbent (g).

## Electronic supplementary material

Supplementary Info
