# Supplementary material for: Removal of cationic and anionic heavy metals from water by 1D and 2D-carbon structures decorated with magnetic nanoparticles
Source: Sci Rep. 2017 Oct 26;7:14107. doi: 10.1038/s41598-017-14461-2 (PMC5658355; doi:10.1038/s41598-017-14461-2)
Supplement: Supplementary file 1 — Supplementary Info [file 41598_2017_14461_MOESM1_ESM.pdf]

Removal of cationic and anionic heavy metals from water by 1D and 2D-carbon structures decorated with magnetic nanoparticles

Chella Santhosh [a, *], Ravi Nivetha [b], Pratap Kollu [c,d], Varsha Srivastava [e], Mika Sillanpää [e], Andrews Nirmala Grace [b,*], Amit Bhatnagar [a, *]

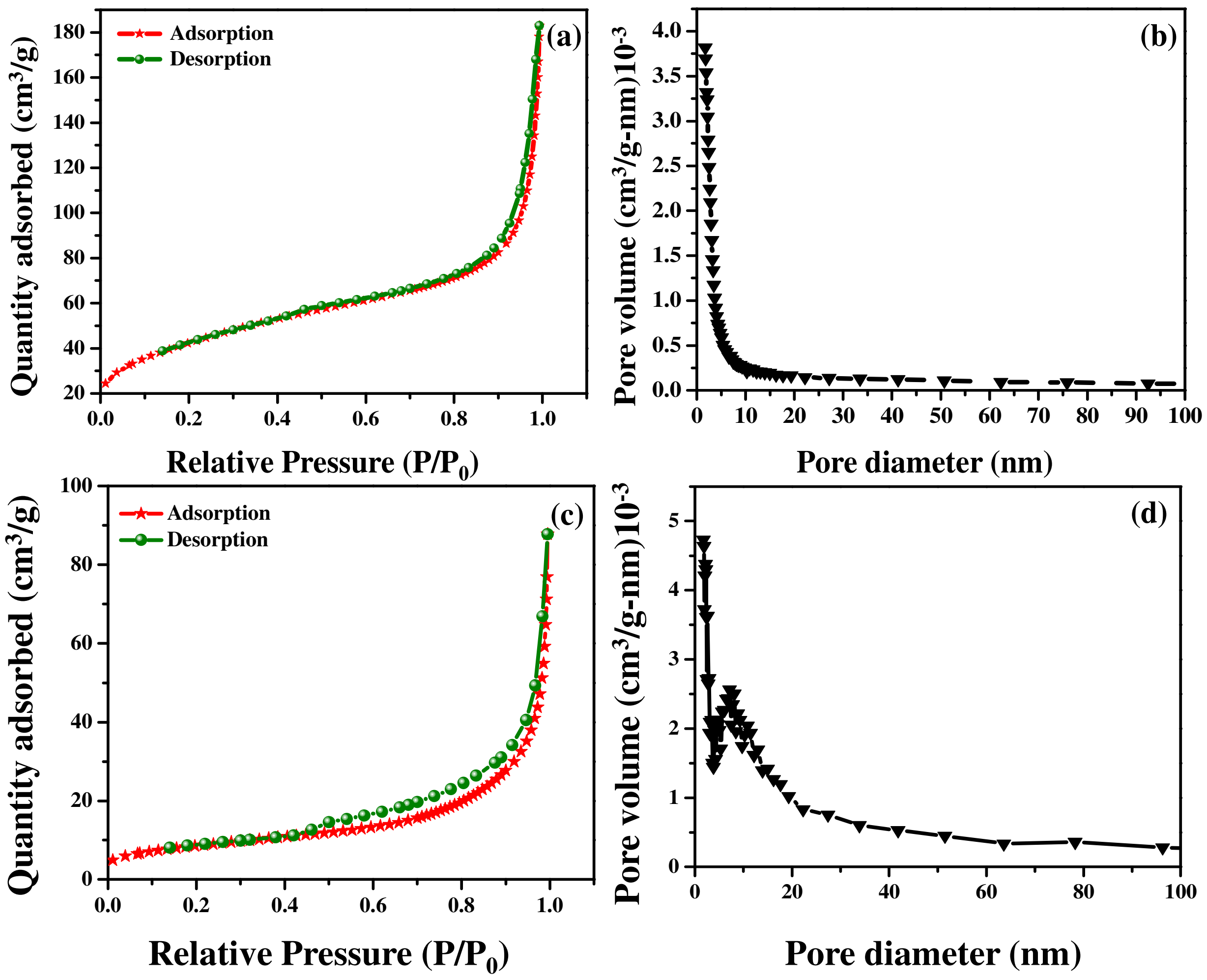

Supplementary Fig. S1. (a, c) N_2 adsorption – desorption isotherm and (b, d) Pore size distribution of PG-C and CNF-C nanocomposites.

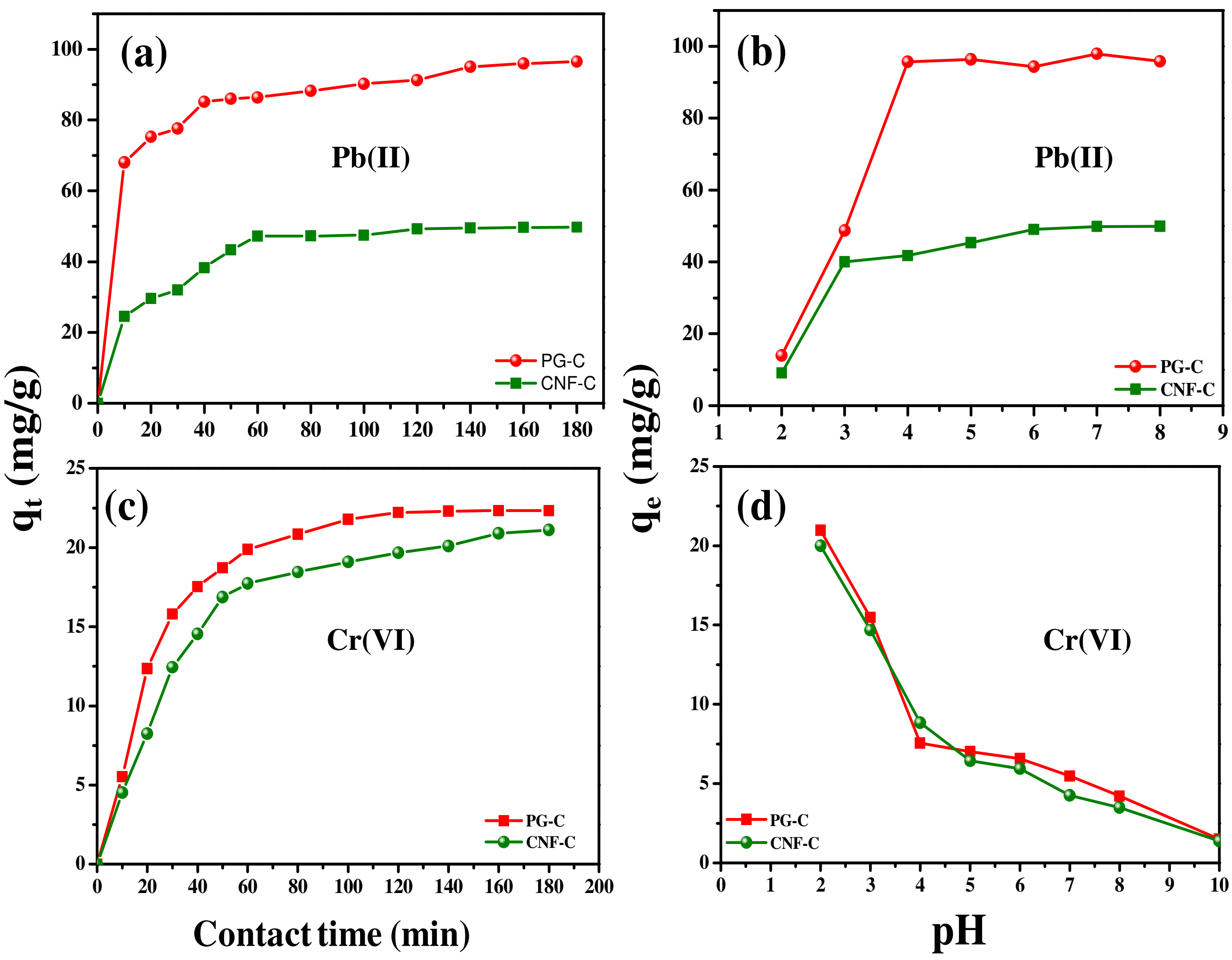

Supplementary Fig. S2. Effect of (a, c) contact time and (b, d) pH of Pb(II) and Cr(VI) ions adsorption by PG-C and CNF-C nanocomposites.

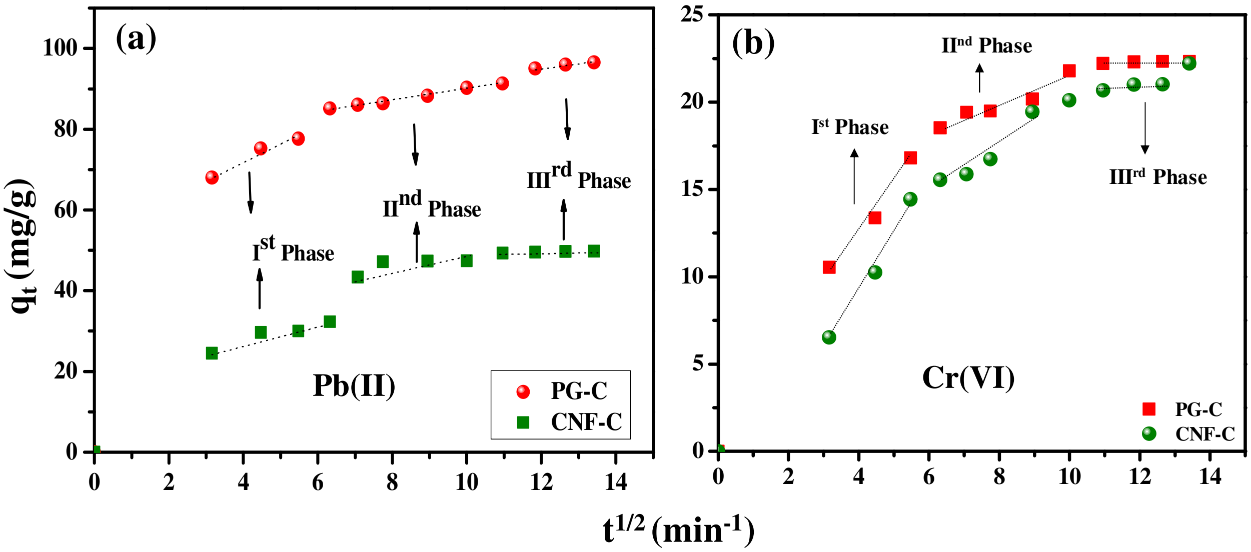

Supplementary Fig. S3. Intra-particle diffusion model for (a) Pb(II) and (b) Cr(VI) ions adsorption by PG-C and CNF-C nanocomposites.

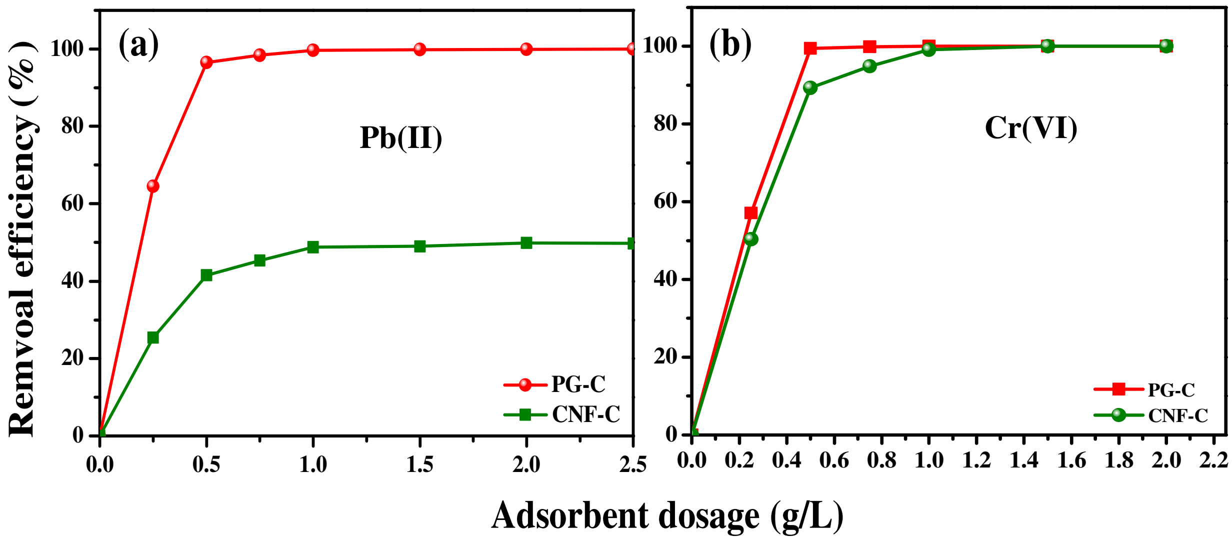

Supplementary Fig. S4. Effect of adsorbent dosage on (a) Pb(II) and (b) Cr(VI) ions adsorption by PG-C and CNF-C nanocomposites.

Supplementary Table S1: Kinetic parameters for Pb(II) and Cr(VI) ions adsorption onto synthesized nanocomposites.

**Adsorbate**	**Adsorbent**	***Pseudo-second-order model***				
		$q_{e(exp)}$ (mg g^{-1})	k_2 (min^{-1})	$q_{e(cal)}$ (mg g^{-1})	RMSE	R^2
Pb(II)	PG-C	96.54	0.002	95.85	2.25	0.998
	CNF-C	49.76	0.001	55.78	2.71	0.992
Cr(VI)	PG-C	22.33	0.004	23.97	6.03	0.990
	CNF-C	21.11	0.002	22.34	5.62	0.991

**Adsorbate**	**Adsorbent**	***Avrami model***					
		$q_{e(exp)}$ (mg g^{-1})	K_{av}	n_{av}	$q_{e(cal)}$ (mg g^{-1})	RMSE	R^2
Pb(II)	PG-C	96.54	0.017	6.78	89.70	4.57	0.994
	CNF-C	49.76	0.006	6.79	49.13	3.16	0.989
Cr(VI)	PG-C	22.33	0.038	1.33	22.21	5.99	0.986
	CNF-C	21.11	0.031	1.53	19.78	5.26	0.832

**Adsorbate**	**Adsorbent**	***Intra-particle diffusion model***				
		C_i (mg L^{-1})	I	K_p	RMSE	R^2
Pb(II)	PG-C	50	5.24	0.25	70.98	0.906
	CNF-C	50	5.24	0.25	31.72	0.956
Cr(VI)	PG-C	10	11.32	1.00	8.86	0.936
	CNF-C	10	11.32	1.00	9.13	0.959

Supplementary Table S2: Isotherm studies for Pb(II) and Cr(VI) ions onto the PG-C and CNF-C nanocomposites.

Adsorbate	Adsorbent	***Langmuir isotherm model***				
		q_m (mg g^{-1})	K_L	R_L	RMSE	R^2
Pb(II)	PG-C	131.40	2.05	0.009	14.75	0.987
	CNF-C	42.90	7.10	0.002	5.19	0.985
Cr(VI)	PG-C	68.85	2.70	0.004	7.84	0.990
	CNF-C	51.07	3.20	0.002	5.87	0.989

Adsorbate	Adsorbent	***Freundlich isotherm model***				
		K_F (mg g^{-1}) (L mg^{-1})$^{1/n}$	n	-	RMSE	R^2
Pb(II)	PG-C	73.91	5.53	-	19.15	0.786
	CNF-C	29.48	9.86	-	9.80	0.510
Cr(VI)	PG-C	33.62	5.55	-	9.64	0.847
	CNF-C	29.73	7.43	-	7.58	0.897

Adsorbate	Adsorbent	***Sips isotherm model***				
		q_m (mg g^{-1})	Ks(L mg^{-1})	n	RMSE	R^2
Pb(II)	PG-C	130.51	2.32	1.07	15.20	0.965
	CNF-C	44.34	5.43	0.85	6.42	0.976
Cr(VI)	PG-C	136.54	0.34	0.27	9.42	0.942
	CNF-C	72.22	0.76	0.29	5.62	0.914

Adsorbate	Adsorbent	***Redlich-Peterson isotherm model***				
		K_{RP} (L mg^{-1})	a	n	RMSE	R^2
Pb(II)	PG-C	238.6	1.77	0.85	15.61	0.858
	CNF-C	30.99	1.58	1.12	6.13	0.803
Cr(VI)	PG-C	207.90	33.00	0.81	9.35	0.887
	CNF-C	107.82	29.85	0.86	7.58	0.897

Supplementary Table S3: Thermodynamic parameters for Pb(II) and Cr(VI) ions onto synthesized nanocomposites.

		**Pb(II)**			**Cr(VI)**		
Adsorbent	Temp. (K)	ΔG° ($kJmol^{-1}$)	ΔH° ($kJmol^{-1}$)	ΔS° ($Jmol^{-1}K^{-1}$)	ΔG° ($kJmol^{-1}$)	ΔH° ($kJmol^{-1}$)	ΔS° ($Jmol^{-1}K^{-1}$)
PG-C	298	-9.96	6.07	1.19	-17.94	15.42	0.45
	308	-9.05			-16.16		
	318	-3.02			-14.73		
CNF-C	298	-13.21	12.45	3.65	-14.01	27.87	0.55
	308	-10.97			-12.31		
	318	-5.76			-8.04		
